# The study protocol of: ‘Initiating end of life care in stroke: clinical decision-making around prognosis’

**DOI:** 10.1186/1472-684X-13-55

**Published:** 2014-12-05

**Authors:** Christopher R Burton, Sheila Payne, Mary Turner, Tracey Bucknall, Jo Rycroft-Malone, Pippa Tyrrell, Maria Horne, Lupetu Ives Ntambwe, Sarah Tyson, Helen Mitchell, Sion Williams, Salah Elghenzai

**Affiliations:** School of Healthcare Sciences, Bangor University, Bangor, Gwynedd LL57 2EF UK; International Observatory on End of Life Care, Faculty of Health and Medicine, Lancaster University, Lancaster, LA1 4YG UK; School of Nursing and Midwifery, Deakin University, 221 Burwood Hwy, Burwood, Melbourne, VIC 3125 Australia; University of Manchester, MAHSC, Salford Royal Foundation Trust, M6 8HD Salford, UK; University of Bradford, Richmond Road, Bradford, Yorkshire, BD7 1DP UK; University of Manchester, Oxford Road, Manchester, M13 9PL UK; Betsi Cadwaladr University Health Board, Ysbyty Eryri, Caernarfon, LL55 2YE UK; Betsi Cadwaladr University Health Board, Ysbyty Gwynedd, Penrhosgarnedd, Bangor, Gwynedd, LL57 2PW UK

**Keywords:** Acute stroke, Palliative care, End of life care, Decision making, Dying trajectories, Implementation

## Abstract

**Background:**

The initiation of end of life care in an acute stroke context should be focused on those patients and families with greatest need. This requires clinicians to synthesise information on prognosis, patterns (trajectories) of dying and patient and family preferences. Within acute stroke, prognostic models are available to identify risks of dying, but variability in dying trajectories makes it difficult for clinicians to know when to commence palliative interventions. This study aims to investigate clinicians’ use of different types of evidence in decisions to initiate end of life care within trajectories typical of the acute stroke population.

**Methods/design:**

This two-phase, mixed methods study comprises investigation of dying trajectories in acute stroke (Phase 1), and the use of clinical scenarios to investigate clinical decision-making in the initiation of palliative care (Phase 2). It will be conducted in four acute stroke services in North Wales and North West England. Patient and public involvement is integral to this research, with service users involved at each stage.

**Discussion:**

This study will be the first to examine whether patterns of dying reported in other diagnostic groups are transferable to acute stroke care. The strengths and limitations of the study will be considered. This research will produce comprehensive understanding of the nature of clinical decision-making around end of life care in an acute stroke context, which in turn will inform the development of interventions to further build staff knowledge, skills and confidence in this challenging aspect of acute stroke care.

## Background

Despite significant advances in the organisation and content of stroke services, nearly 20% of patients will die in the acute phase; 11% of all deaths in England and Wales are due to stroke [1]. Stroke services are required to ensure that best end of life care, usually defined within a cancer context, is implemented [2]. However, the rehabilitation practice context may seem at odds with palliative care, and the evidence base to guide this in stroke is lacking [3]. In a previous study to address a lack of prospective information on patient and family needs, we demonstrated that acute stroke patients have a high burden of problems that the principles of palliative care can address [4]. For example, in a consecutive sample of acute stroke admissions, over 50% reported moderate to significant fatigue-related problems, symptom-related problems such as pain, or psychological distress. Approximately one in every four patients had concerns about death or dying.

Although knowledge of factors that predict acute stroke mortality is available [5], recognition of a stroke patient’s ‘dying’ status may be ambiguous [6], potentially resulting in over or under treatment and delaying initiation of general palliative care or referral to specialist palliative care. Older people may be disadvantaged when accessing appropriate and acceptable services that meet their preferences and those of their family carers, who may also be older people [7]. A specific challenge is the initiation of end of life care [6]. There are problems in recognising the process of dying and assigning an entry point to ‘end of life’ is always going to be somewhat arbitrary [8]. Hypothesised models of typical dying trajectories linked to cancer, organ failure and frailty have not always been supported by empirical data [9, 10]. Earlier models have assumed a linear trajectory of decline [11], or have focused on psychosocial transitions [12] alone. More recently other models have been proposed to capture the fluctuations in palliative care input relative to other healthcare interventions, including curative treatment [13, 14], reinforcing the need for a seamless integration of palliative and end of life care within the acute stroke pathway.

Inevitably, acute stroke onset presents a significant threat to patients and families, and these impacts are well documented in the literature. Although most patients survive acute stroke, patients and their families have concerns about death and dying that do not appear to be related to prognosis [6]. Opportunities to discuss and help make sense of these concerns may be important, and any uncertainty about prognosis should not prevent these issues from being explored. Dealing with end of life issues places considerable demands on patients and family members; the role of clinicians in this context is to initiate timely and effective support to help patients and families cope with and adapt to these demands. Policy and clinical guidance both highlight the importance of information provision, communication and decision-making within a multi-disciplinary context, and partnership with patients and families to determine care preferences [15, 16]. However, the implementation of evidence-based guidelines around palliative and end of life care in stroke is complex [17], with staff-related challenges including professional beliefs about legitimacy of palliative care; confidence and skills; engaging with family members; recognising dying and knowing when to commence end of life care interventions.

The major emphasis of acute stroke care is on ensuring neurological recovery or stability, preventing complications and commencing early rehabilitation [18]. Patients and families also require access to professional interventions that ameliorate negative disease sequelae, and support them during a potentially life threatening event. However, the evidence base for the effectiveness of supportive strategies to address these issues in stroke is diffuse, and lacking in any theoretical integrity [19]. There appears to be a dilemma for staff when considering the nature and purpose of end of life care in acute stroke services, including whether end of life care focuses predominantly on death, or more supportive interventions that could (or not) be combined with active treatment strategies to improve the quality of an individual’s limited life. A discourse around 'supportive care' may provide an acceptable middle-ground where staff could see the value of palliative care in promoting 'quality of life' rather than merely 'a good death'.

Reflecting on frameworks from the implementation literature (e.g. [20]), the use of information in end of life care is a function of the interplay between the different types of evidence used by clinicians to inform decisions; individual and organisational influences on use; and the tools and strategies through which use is enabled. Dying trajectories in acute stroke, prognostic factors associated with each trajectory, and service user preferences for the initiation of end of life care within and across trajectories provide a mix of evidence to guide clinical practice [21]. Decision science studies the cognitive processes underpinning the selection of evidence, specifically the filtering and integration of current scientific information into changing contexts [22]. However, Downie and Macnaughton [23] suggest that scientific facts only become evidence when the clinician decides the information is relevant to a particular case. In Hogarth’s [24] model of decision making, individual characteristics such as personal attributes, values and capabilities, the complexity of the task and the external environment all shape the behaviour of the decision maker. This study will provide a novel opportunity to investigate the different types of evidence used by individual clinicians in making decisions on end of life care. The outcomes of the study will inform our understanding of end of life decision making and enhance the transferability of findings to other, related diagnostic groups.

### The study

#### Aims and objectives

The overall aim of the study is to investigate clinicians’ use of different types of evidence in decisions to initiate end of life care within trajectories typical of the acute stroke population.

The specific objectives are:

- ● To identify trajectories of dying in acute stroke;
- ● To investigate patients’ and family members’ experiences of the initiation of end of life care within acute stroke;
- ● To identify how clinicians draw on different types of prognostic, clinical and other information sources used in making decisions about initiating end of life care in acute stroke; and
- ● To determine the influences on clinical decisions concerning end of life care in acute stroke.

## Methods/Design

This is a two-phase, mixed methods study comprising investigation of dying trajectories in acute stroke (Phase 1), and the use of clinical scenarios to investigate clinical decision-making in the initiation of end of life care (Phase 2). We will be guided by a steering group comprising the research team and collaborators, with representation from regional Patient and Public Involvement (PPI) groups.

### Service user involvement

We have consulted and will continue to consult with PPI representatives and with people living with stroke in North West England and North Wales. Consultation includes issues around the study ‘language’, the acceptability of recruitment processes, and the content and structure of interview schedules. In addition, two members of the Lancaster Research Partners Forum (a group of service users with training in research appreciation) have joined the project team.

### Phase 1

This phase is an observational patient tracking study to identify dying trajectories within acute stroke, with interviews of a sub-sample of patients and/or carers and bereaved carers to explore views about the initiation of end of life care. For the purposes of this study, acute stroke is defined as the first 28 days after stroke onset [25]. Patterns of dying across a range of conditions have been described qualitatively, but have yet to be specified in an acute stroke population. Our starting point for the development of dying trajectories will be an adaptation of the four trajectories identified by Lunney et al. [9] and proposed for use in non-malignant diseases [26]. This typology is underpinned by a grounded theory study [27], and has been used for the retrospective classification of dying trajectories across a large sample of older decedents in the US. We will use the same sequential profiling methods as Lunney et al. [9] to allocate decedent patients to an individual trajectory, although the clinical markers we will use are different, and will be validated by the research team and study collaborators.

Initially, patient deaths will be classified by the lead stroke clinician as Trajectory 1 or not. Where there is evidence of a distinct terminal phase, then deaths will be classified as Trajectory 2. For remaining patient deaths, where there is evidence of multiple professional inputs designed to address a specific problem, then these will be classified as Trajectory 3. The remaining deaths will be classified as Trajectory 4, or ‘other’ where it is not possible to classify using the textual descriptions of each Trajectory type (see Table 1).

**Table 1:** **Typology of potential dying trajectories in acute stroke**

		Markers from Lunney et al. 2002 [9]	Proposed typology
1	Sudden	Little evidence of healthcare in the last year of life	Unexpected death - e.g. ineffective resuscitation; complications of thrombolysis; overwhelming cardio/cerebrovascular event
2	Rapid death	Plurality of physician input in last year of life	Expected death; distinct terminal phase although not set within a general context of deterioration
3	Episodic	Evidence of multiple acute interventions; exacerbations of health problems; evidence of organ failure	Evidence of increasing health problems; multiple acute, curative interventions; indistinct terminal phase
4	Slow decline	Frailty associated with long-term health condition	Multiple health problems; general picture of persisting, and overwhelming illness
5	Other		Unable to classify as above

### Setting and participants

The first phase of the study is being conducted over a six month period in four acute stroke services (three in North Wales and one in North West England). We aim to include 832 patients, and estimate that 141 of these patients may die in the acute stroke phase. In addition, semi-structured interviews of a sub-sample of up to 30 patients, family carers and bereaved relatives will uncover associated chronological narratives of experience.

### Inclusion criteria

All patients with a clinical diagnosis of acute stroke, confirmed by a lead clinician through entry into the acute stroke register, will be eligible for inclusion in the study. Patients presenting with subarachnoid haemorrhage, or who are unable to consent with no personal consultee, will be excluded. Individuals that self-identify as family carers of a patient will be able to participate in interviews.

### Data collection

Soon after admission, potential participants and their family members will be introduced to the study by a member of the clinical team, and provided with a study information pack. This pack includes information about the purpose of the study and details of what will happen to them if they decide to take part. If patients and/or family members are interested in participating, they will be asked to leave an expression of interest form in a sealed envelope with a member of the clinical team. For those patients who wish to take part, a member of Research Network staff will meet with them in hospital to take their consent. Once consent/consultation has been recorded, the patients’ GP will be informed of their participation in the study.

For patients unable to consent for themselves, a family member able to advise on the presumed wishes of the patient will be approached to act in the role of consultee. This is in line with the recommendations of the Mental Capacity Act 2005 [28] and also with the expressed wishes of patient and public involvement representatives, who would like everyone who is eligible to have the opportunity to participate.

Study registers will be maintained at each study site by Research Network staff. These will extract the minimum data outlined in Table 2 for every acute stroke admission from each hospital’s database of stroke admissions. This will allow a minimum amount of non-identifiable data to be collected to enable the mapping of different dying trajectories. For patients that consent, a range of biographical and clinical information will be extracted from the patients’ hospital records. This will include information about them and their family, their health, and the care they received whilst in hospital or from their GP. In addition, the research staff will also complete an assessment of the dying trajectory for patients that die within the acute stroke phase.

**Table 2:** **Phase 1 data collection summary**

All eligible patients	Consented patients	
Patient age at stroke event	Name	***Interviews with up to 10 consented patients:***
Gender	Address	
Date of stroke event	Contact details	Date of interview
Stroke subtype	Marital status	Interviewer reflections on interview
Date of admission	Living accommodation	
Date and destination of transfer of care	Presence of family carer	Interview transcript
Date and primary/secondary causes of death date of admission	***On admission:***	***Interviews with up to 5 bereaved family relatives:***
	Presence of Advance Care Plan	
Dying trajectory type (completed by clinical staff)	Co-morbidities (Number)	Date of interview
	Modified Rankin	Interviewer reflections on interview
	Barthel Index	
	Communication	Interviewer transcript
	Cognition	
	***During acute stroke phase:***	
	Number and type of palliative or end of life referrals, and evidence of referrals implemented	
	Use of DNR orders, including date of discussion with family/implementation	
	Use of End of Life Pathway, including start date	
	Dying trajectory type (completed by research and clinical staff)	

Some participants will be invited to take part in an interview to explore their experiences and preferences for care. Interviews will draw on individually meaningful markers to identify the initiation of end of life care (e.g. information provision, withdrawal of interventions), and the way that this impacts upon experiences of care and illness progression. These interviews will be conducted in hospital or at home, dependent on participant preference, and may include a family carer if the patient wishes, or has additional communication support needs. We will also interview a small number of family members of patients who have died from their acute stroke. As with the National Bereavement Survey [29], we will not contact family members in these circumstances until at least three months after the death. These interviews will be conducted by an experienced palliative care researcher in the participant’s home or other place of their choosing.

### Data analysis

The data will be used to construct typologies of ‘patterns of dying’ or trajectories after stroke. We will use these typologies to develop case scenarios for a subsequent investigation of clinical decision-making around end of life care.

Quantitative data will be analysed using SPSS software. Analysis will commence with the production of descriptive statistics (central tendency and variation for interval data; frequencies and percentages for categorical data) and appropriate graphical summaries for recruitment and consent rates, and participant characteristics across each clinical site. T-tests or Chi Square tests will be used where relevant to identify important differences in the samples across each clinical site. The remainder of the analyses will be conducted on the dataset as a whole.

The main analyses will focus on the detailed investigation of patients who die within the acute stroke phase. The purpose of the analysis is not to evaluate any of the prognostic models for acute stroke mortality that can be found in the literature. Rather it is to provide an integrated overview of the different dying trajectories which may be observed.

Initially the inter-rater reliability between study and clinical staffs’ assessment of dying trajectories will be assessed using the kappa statistic [30]. For subsequent analyses, only the assessment of trajectory type by clinical staff will be used. The analyses will include a description of each trajectory, including where possible the production of Kaplan Meier curves for the subset of patients who died within each of the trajectories. Univariate analyses of key patient, clinical and service variables across each trajectory type will be performed. The final analytical task will be to produce a narrative of the different trajectories which may be observed. This narrative will juxtapose patient and clinical variables and summaries of service provision with data on the time to death and cause of death through the use of charts [31].

Interview transcripts, interviewer reflections and any field notes will be managed in NVivo software. Commonalities and differences within individual accounts (patients, current family carers and bereaved relatives) and across the four acute stroke services will be identified. The analysis will be iteratively influenced by the literature and the outcomes from the observational study to maximise interpretive depth, and ensure that the diversity of dying trajectories in stroke is fully addressed. An initial framework of thematic categories will be applied to interview data drawing on the research objectives. Constant comparison will be used so that the early stages of analysis inform subsequent data collection. The aim of the analysis will be to produce insights relating to the research questions which are grounded in the experiences and understandings of the participants, and which are capable of theoretical or logical generalisation.

We will explore the extent to which findings from the thematic analysis and the observational dataset analysis are congruent. We will use both findings to postulate dying trajectories in stroke comprising detailed descriptions of patient, family and clinical perspectives.

### Phase 2

This phase will describe the clinical decisions about end of life care using a ‘think aloud’ approach [32]. This technique examines the information used by individuals when making clinical decisions, providing insight into factors that influence decisions, together with the final judgements and decisions made by an individual. Participants will also be asked to complete a second, follow-on interview to explore how clinicians make decisions about initiating end of life care, the information that they use, and the influences on these decisions.

### Setting and participants

Up to 30 clinicians from stroke units in four hospitals in North Wales and North West England will take part. Participants will reflect a mix of professional roles, experience and qualifications; however, recruitment will focus on clinicians who have responsibility for decision-making within the multidisciplinary team. This is most likely to be doctors, clinical nurse specialists and senior ward staff, but might also include senior allied health professionals. Participants will be purposively sampled from each stroke service.

### Recruitment

The research team already has established links with stroke physicians and specialist nurses at each hospital site, and with the assistance of these individuals the research team will compile a list of key members of the multidisciplinary stroke team at each site.

Following ethical and governance approvals, stroke team members will be provided with information packs about the study and asked to distribute them amongst their colleagues. The packs will include an invitation letter, study information sheet, expression of interest form, consent form and return envelope. Potential participants will be asked to complete the expression of interest form and return it, either by post in the envelope provided or by email, to the research team. A researcher will then contact the participant and arrange a mutually convenient time and place for interviews to take place.

### Data collection

#### Think-aloud exercise

The ‘think-aloud exercise’ will use a clinical scenario, a standard method in decision-making research, to examine the prognostic and other information and cognitive processes that staff use when making judgements and decisions [33].

To begin with, participants will be taken through a practice exercise: they will be asked to visualise walking into their home through their front door, and then asked to describe the route they would take whilst counting the number of windows in each room. Participants will then be given a decision task and asked to ‘think aloud’ or verbalise everything they are thinking whilst carrying out the task [34]. Critical to this is the development of the decision task: we will use case scenarios to represent the types of patients and decision scenarios that clinicians are faced with in initiating end of life care. These case scenarios or ‘vignettes’ will be developed from Phase 1 data and from the clinical expertise of the project team to resemble as closely as possible the types of information clinicians would normally have access to [35, 36]. Case scenarios will be tested for face and content validity with an expert panel (n = 5) experienced in end of life care in acute stroke identified through the UK Stroke Research Network. Participants will be asked to think aloud whilst developing a management plan for the patient described in the scenario. These verbalisations will be recorded and transcribed. To compare decision making across participants, the same set of clinical scenarios will be used.

Prompts used in the think-aloud exercise will focus on participants’ thinking rather than actions per se. Where more information is requested, then the nature of information required, and how this might influence thinking will be explored, and where required, the scenario will be used to shift discussion from the general to the specific. The scenario will not make explicit reference to end of life care. If this is not mentioned by participants, then the interviewer will ask the question, “Would you be thinking that this patient might die?” to complete the think aloud exercise.

#### Semi-structured interviews

All participants in the think-aloud exercise will subsequently be interviewed to explore in more detail their perceptions about how they make decisions about initiating end of life care, and the information that they think they use, including specific challenges they currently face in practice. Participants will be asked to expand on management plans they developed during the think-aloud exercise to provide a detailed exploration of the factors influencing their decision making. Again, all interviews will be digitally recorded and transcribed.

As it is expected that the ‘think-aloud’ exercise will take around 30 minutes to complete, participants will be offered the option of taking part in the interview on another day and/or by telephone, in order to minimise the burden on participants. If preferred, the interview could be run as a focus group with different members of the multidisciplinary team taking part together.

#### Data analysis

Think-aloud study data will be analysed using protocol analysis, an established technique for identifying the information and specific cognitive processes that individuals have used to make decisions [34]. It involves the categorisation of each element of verbalisation into mutually exclusive categories corresponding to a cognitive process (e.g. information seeking). After this analysis, it is possible to analyse different elements of the judgement and decision process (e.g. the information used to inform the decision) and to map the order in which elements of the decision process occur [36]. The analysis will enable us to compare decision making across clinicians, areas of consensus and disagreement.

Interview data will be analysed using thematic analysis [37]. Transcripts from the interviews will be read to identify themes or categories to code the data, and then examined to explore interconnections between themes [38]. The results of the analysis from the interview study will then be synthesised with the protocol analysis providing a detailed insight into how staff use information, and the factors that influence their decision making about initiating end of life care. The analysis will identify patterns of similarity and differences across individuals and staff groups to explore implementation issues.

#### Ethical considerations

Research into end of life care can raise many ethical and professional challenges, particularly around how research is presented to potential participants. Ethical approval for Phase 1 of the study was given by the National Institute for Social Care and Health Research (NISCHR) North Wales Research Ethics Committee West on 26 March 2013 (reference 13/WA/0086) and two substantial amendments to add further measures to the protocol were approved on 11 July 2013 and 3 February 2014. Approval for Phase 2 was given by the Healthcare and Medical Sciences Academic Ethics Committee at Bangor University on 22 May 2014. Written informed consent will be obtained from all study participants (or their consultees). The team have considerable experience in addressing ethical challenges in end of life care and stroke research, including conducting and supervising interviews that may potentially be distressing for patients and families. Recruitment and data collection processes have been designed to ensure that we place no additional stress on patients and families. This includes the use of a ‘neutral’ study title that does not use words such as ‘palliative’ that have loaded meaning within public discourse. To mitigate the potential for emotional burden, we will provide a supportive environment for research staff. Excellent communication skills around stroke-related communication problems can be addressed through the use of aphasia-friendly materials and the use of communication aids within interviews.

Patient and public involvement is an important mechanism in confirming the acceptability of study documentation. However, we have drawn on the skills of our patient and public representatives in exploring the practical issues of approaching patients and families for consent or consultation with research staff. In this way, we hope to ensure that recruitment is maximised, with limited potential for clinician ‘gate-keeping’ to protect those participants perceived as vulnerable by staff [39].

#### Dissemination

Prognostic information on dying and understanding clinicians’ decision-making will maximise the implementation potential for initiating end of life care in acute stroke. We plan to prepare abstracts for submission to stroke (e.g. UK Stroke Forum) and palliative care conferences (e.g. European Association for Palliative Care research conference) and to a range of professional and academic journals. We will use EAPC blogs and stroke improvement resources (e.g. UK Forum for Stroke Training) to rapidly highlight study recommendations to clinicians.

## Discussion

Information on prognostic factors may alert staff to those patients who are at higher risk of death in the acute stroke phase. However, this information alone may be insufficient to enable staff to recognise impending death, and to plan care that meets the preferences of patients and families in these circumstances. Rapid and unexpected deaths provide little opportunity for staff to implement anything other than palliative care at the very end of life. Understanding of the complete range of patterns of dying and survival may equip stroke service staff with the knowledge and confidence to weave palliative and end of life interventions into a patient’s programme of care.

This study will be the first to examine whether patterns of dying reported in other diagnostic groups are transferable to acute stroke care. In addition the study will explore the process of clinical decision-making in this area. The study is located within the decision-making and implementation literature, focusing on the importance of context, and the breadth of evidence and information that staff may draw on to guide their clinical practice. Our intention is that an in-depth understanding of the nature of clinical decision-making, coupled with evidence of observable patterns of dying, will inform the development of interventions to further build staff knowledge, skills and confidence in this challenging aspect of acute stroke care.

The strengths of this study lie in the mix of methods, which will simultaneously generate evidence of dying trajectories, and the nature of associated clinical decision-making by staff. Integrating both quantitative and qualitative perspectives on patterns of dying will provide a comprehensive overview on which to base further research. In addition, the mix of academic and professional disciplines, together with a service user voice, will ensure that the implementation of this study and the interpretation of findings are robust.

We should draw attention to some of the challenges that have been experienced in setting up this study, or that are anticipated. The study spans health services in both England and Wales; both countries have separate research governance approval strategies and processes. Although these are intended to dovetail as much as possible, the review requirements of each have complicated the study set-up process. Attention has been paid to strategies that enable participation in the study in ways that maximise the collection of a minimum of data with minimal inconvenience to patients. The challenge of enabling the participation of patients with more severe strokes, and who may be at greater risk of death, should not be underestimated. Permission has been granted to obtain routine, anonymised data from all acute stroke admissions, with consent (or consultation) required for the collection of only additional data. However the collection of these data, which may represent factors associated with the differing patterns of dying, which themselves may vary in incidence, will depend on the ability of research network staff to approach patients and families in a timely manner.

This research will produce a comprehensive overview of the nature of clinical decision-making around end of life care in an acute stroke context. We aim to provide clinicians with an understanding of the patterns of dying in acute stroke, together with patient and family experiences of end of life care. This will complement the available statistical models which are prognostic of acute stroke mortality. Our hypothesis is that this combination of evidence will improve quality in this challenging area of practice. As interviews with staff will also explore the context around the implementation of end of life care guidance more generally, it is hoped that the findings of this investigation will inform new workforce development interventions to enhance the confidence and skills of acute stroke staff in providing palliative and end of life care.

## References

[1] Royal College of Physicians (2011). National Sentinel Stroke Clinical Audit 2010 Round 7.

[2] Department of Health (2007). National Stroke Strategy.

[3] Stevens T, Payne SA, Burton C, Addington-Hall J, Jones A (2007). Palliative care in stroke: a critical review of the literature. Palliat Med.

[4] Burton CR, Payne S, Addington-Hall J, Jones A (2010). The palliative care needs of acute stroke patients: a prospective study of hospital admissions. Age Ageing.

[5] Counsell C, Dennis M (2001). Systematic review of prognostic models in patients with acute stroke. Cerebrovasc Dis.

[6] Payne S, Burton C, Addington-Hall J, Jones A (2010). End-of-life issues in acute stroke care: a qualitative study of the experiences and preferences of patients and families. Palliat Med.

[7] Seymour J, Witherspoon R, Gott M, Ross H, Payne S (2005). Dying in Older Age: End-of-Life Care.

[8] Glare P, Christakis NA, Glare P, Christakis NA (2008). Overview: Advancing the Clinical Science of Prognostication. Prognosis in Advanced Cancer.

[9] Lunney JR, Lynn J, Hogan C (2002). Profiles of older medicare decedents. J Am Geriatr Soc.

[10] Gott M, Barnes S, Parker C, Payne S, Seamark D, Gariballa S, Small N (2007). Dying trajectories in heart failure. Palliat Med.

[11] Lynn J, Adamson DM (2003). Living Well at the End of Life. Adapting Health Care to Serious Chronic Illness in Old Age.

[12] Glaser BG, Strauss AL (1965). Awareness of Dying.

[13] Gott M, Small N, Barnes S, Payne S, Seamark D (2008). Older people’s views of a good death in heart failure: Implications for palliative care provision. Soc Sci Med.

[14] Payne S, Froggatt K, O’Shea E, Murphy K, Larkin P, Casey D, Léime A (2009). Improving palliative and end-of-life care for older people in Ireland: a new model and framework for institutional care. J Palliat Care.

[15] General Medical Council (2007). Treatment and Care Towards the end of Life: Good Practice in Decision Making.

[16] Department of Health (2010). Equity and Excellence: Liberating the NHS.

[17] Burton C, Payne S (2012). Integrating palliative care within acute stroke services: developing a programme theory of patient and family needs, preferences and staff perspectives. BMC Palliat Care.

[18] National Institute for Health and Clinical Excellence (2008). Diagnosis and Initial Management of Acute Stroke and Transient Ischaemic Attack (TIA).

[19] Lerdal A, Bakken LN, Kouwenhoven SE, Pedersen G, Kirkevold M, Finset A, Kim HS (2009). Post-stroke fatigue – a review. J Pain Symptom Manag.

[20] Rycroft-Malone J, Harvey G, Seers K, Kitson A, McCormack B, Titchen A (2004). An exploration of the factors that influence the implementation of evidence into practice. J Clin Nurs.

[21] Bamford J, Sandercock P, Dennis M, Warlow C, Burn J (1991). Classification and natural history of clinically identifiable subtypes of cerebral infarction. Lancet.

[22] Bucknall TK (2007). A gaze through the lens of decision theory towards knowledge translation science. Nurs Res.

[23] Downie R, Macnaughton J (2000). Clinical Judgement.

[24] Hogarth RM (1980). Judgement and Choice: The Psychology of Decision.

[25] Roberts SE, Goldacre MJ (2003). Case fatality rates after hospital admission for stroke. BMJ.

[26] Murtagh FEM, Preston M, Higginson I (2004). Patterns of dying: palliative care for non-malignant disease. Clin Med.

[27] Glaser B, Strauss AL (1968). A Time for Dying.

[28] *Mental Capacity Act*. 2005. [http://www.legislation.gov.uk/ukpga/2005/9/contents]

[29] National Bereavement Survey: *National Bereavement Survey*. [http://www.ons.gov.uk/ons/rel/subnational-health1/national-survey-of-bereaved-people--voices-/2013/index.html]

[30] Fleiss JL (1971). Measuring nominal scale agreement among many raters. Psychol Bull.

[31] Miles MB, Huberman AM (1994). Qualitative Data Analysis: An Expanded Sourcebook.

[32] Bucknall TK, Aitken LM, Gerrish K, Lacey A (2009). Think Aloud Technique. The Research Process in Nursing.

[33] Thompson C, Dowding D (2001). Clinical Decision Making and Judgement in Nursing.

[34] Fonteyn ME, Kuipers B, Grobe SJ (1993). A description of think aloud method and protocol analysis. Qual Health Res.

[35] Skanér Y, Strender L-E, Bring J (1998). How do GPs use clinical information in their judgements of heart failure?. Scand J Prim Health Care.

[36] Lamond D, Crow R, Chase J (1996). Judgements and processes in care decisions in acute medical and surgical wards. J Eval Clin Pract.

[37] Ritchie J, Spencer L, Bryman A, Burgess R (1994). Qualitative Data Analysis for Applied Policy Research. Analysing Qualitative Data.

[38] Pope C, Ziebland S, Mays N, Pope C, Mays N (2006). Analysing Qualitative Data. Qualitative Research in Health Care.

[39] Hudson P, Aranda S, Kristjanson L, Quinn K (2005). Minimising gate-keeping in palliative care research. Eur J Palliat Care.

[CR40] The pre-publication history for this paper can be accessed here: http://www.biomedcentral.com/1472-684X/13/55/prepub

